# Krüppel-Like Factor 2 Is Required for Normal Mouse Cardiac Development

**DOI:** 10.1371/journal.pone.0054891

**Published:** 2013-02-14

**Authors:** Aditi R. Chiplunkar, Tina K. Lung, Yousef Alhashem, Benjamin A. Koppenhaver, Fadi N. Salloum, Rakesh C. Kukreja, Jack L. Haar, Joyce A. Lloyd

**Affiliations:** 1 Department of Human and Molecular Genetics, Virginia Commonwealth University, Richmond, Virginia, United States of America; 2 Department of Internal Medicine, Virginia Commonwealth University, Richmond, Virginia, United States of America; 3 Department of Anatomy and Neurobiology, Virginia Commonwealth University, Richmond, Virginia, United States of America; 4 Massey Cancer Center, Virginia Commonwealth University, Richmond, Virginia, United States of America; Brigham and Women’s Hospital, Harvard Medical School, United States of America

## Abstract

Krüppel-like factor 2 (KLF2) is expressed in endothelial cells in the developing heart, particularly in areas of high shear stress, such as the atrioventricular (AV) canal. KLF2 ablation leads to myocardial thinning, high output cardiac failure and death by mouse embryonic day 14.5 (E14.5) in a mixed genetic background. This work identifies an earlier and more fundamental role for KLF2 in mouse cardiac development in FVB/N mice. FVB/N KLF2−/− embryos die earlier, by E11.5. E9.5 FVB/N KLF2−/− hearts have multiple, disorganized cell layers lining the AV cushions, the primordia of the AV valves, rather than the normal single layer. By E10.5, traditional and endothelial-specific FVB/N KLF2−/− AV cushions are hypocellular, suggesting that the cells accumulating at the AV canal have a defect in endothelial to mesenchymal transformation (EMT). E10.5 FVB/N KLF2−/− hearts have reduced glycosaminoglycans in the cardiac jelly, correlating with the reduced EMT. However, the number of mesenchymal cells migrating from FVB/N KLF2−/− AV explants into a collagen matrix is reduced considerably compared to wild-type, suggesting that the EMT defect is not due solely to abnormal cardiac jelly. Echocardiography of E10.5 FVB/N KLF2−/− embryos indicates that they have abnormal heart function compared to wild-type. E10.5 C57BL/6 KLF2−/− hearts have largely normal AV cushions. However, E10.5 FVB/N and C57BL/6 KLF2−/− embryos have a delay in the formation of the atrial septum that is not observed in a defined mixed background. KLF2 ablation results in reduced Sox9, UDP-glucose dehydrogenase (Ugdh), Gata4 and Tbx5 mRNA in FVB/N AV canals. KLF2 binds to the Gata4, Tbx5 and Ugdh promoters in chromatin immunoprecipitation assays, indicating that KLF2 could directly regulate these genes. In conclusion, KLF2−/− heart phenotypes are genetic background-dependent. KLF2 plays a role in EMT through its regulation of important cardiovascular genes.

## Introduction

Congenital heart defects (CHDs) are a leading cause of infant morbidity and mortality (reviewed in [1]). Valve and septal defects account for the majority of CHDs. Mutations in transcription factor genes, including Nkx2-5 [2] and the cardiac T-box gene TBX5 [3], are important for normal valve development in humans. Mutations or variants in other transcription factor genes are likely to be involved in valve defects.

During mouse heart development, localized swellings of the endocardial layer arise at approximately embryonic day 9.5 (E9.5), and form the endocardial cushions of the atrioventricular (AV) canal and the outflow tract. The endocardial cushions are formed by endothelial to mesenchymal transformation (EMT). During EMT, AV endocardial cushion cells undergo hypertrophy, loss of cell-cell contacts, lateral mobility, formation of mesenchymal-like cell processes (filopodia), and migration into the cardiac jelly (Reviewed in [4]). Without normal cardiac jelly, endothelial cells fail to transform and to migrate, resulting in hypoplastic endocardial cushions [5]. Extensive remodeling and proliferation of the endocardial cushions occurs to form the adult heart valves. By E10.5, the right and left atria have divided [6]. The AV endocardial cushion region also plays an important role in septation of the heart (Reviewed in [7]).

Krüppel-like factor 2 (KLF2) is a member of a family of zinc finger-containing transcription factors [8]–[10]. In the E9.5 mouse heart, KLF2 mRNA is highly expressed in the endocardial cells of the AV cushion regions [11], where it is likely induced by high shear stress [12]. The role of KLF2 in cardiac development was studied by Lee et al., in knockout (KO) mice in a mixed genetic background including C57BL/6J. In KLF2 KO and KLF2 endothelial conditional KO embryos, a cardiac phenotype is observed as early as E10.5. These mice have thinning of the myocardium, high output heart failure, and die by E14.5. These KLF2−/− embryos also have cardiac functional defects due to loss of vessel tone, but no role for KLF2 in cushion formation was noted [11]. In two other KLF2−/− models, embryos in a mixed genetic background die between E12.5 and E14.5, and exhibit hemorrhaging [1], [13]. The zebrafish gene, klf2a, a homolog of KLF2 in mouse and man, is important in valve development. Knockdown of klf2a causes thicker and less flexible valves and increased regurgitation in the developing heart, compared to WT [14].

In tissue culture, KLF2 plays a role as a molecular transducer of fluid shear forces, thus directly or indirectly regulating a number of endothelial genes [15], [16]. Recent findings suggest that KLF2 plays an important role in endothelial barrier function in adult mice. It positively regulates expression of the tight junction protein occludin and modification of myosin light chain that is important for the integrity of the endothelial layer and to avoid vascular leakage [17]. In adult mice, KLF2 positively regulates the expression of vasoprotective genes and inhibits expression of pro-inflammatory genes in endothelial cells and macrophages, preventing atherosclerosis [18].

klf2a knockdown results in abnormal zebrafish heart valve development, and KLF2 is expressed in the mouse endocardial cushion region. This suggests that KLF2 may be important in the early stages of mammalian valve development. In this work, we show that FVB/N KLF2−/− hearts are hyperplastic with respect to cells lining the AV canal, and hypoplastic with respect to endocardial cushion mesenchymal cells. The data suggests that KLF2 regulates EMT, and also atrial septation. KLF2 activates multiple important cardiovascular development genes, suggesting mechanisms for its roles in the embryonic heart.

## Materials and Methods

### Ethics Statement

This study was approved by the Virginia Commonwealth University Institutional Animal Care and Use Committee (VCU IACUC). VCU IACUC Protocol Number: AM10347.

### Generation of Knockout and Transgenic Mice

The traditional KLF2 KO mouse model was developed by targeting the gene with the hypoxanthine phosphoribosyl-transferase (Hprt) gene [13]. KLF2+/− adults were mated with FVB/N or C57BL/6 mice for at least 12 generations to obtain KLF2+/− animals in the FVB/N or C57BL/6 genetic background. These animals were then mated to obtain KLF2−/− embryos. FVB/N and C57BL/6 KLF2+/− mice were mated to obtain KLF2+/− animals in a 50% FVB/N and 50% C57BL/6 genetic background (mixKLF2+/−). mixKLF2+/− adults were mated to obtain mix WT and mix KLF2−/− embryos.

Tie2-cre transgenic animals were purchased from Jackson Research Laboratories (Bar Harbor, ME). Mice with a KLF2 allele surrounded by loxP sites (floxed allele or KLF2^fl/+)^ were kindly provided by Dr. Jerry Lingrel, University of Cincinnati and were generated as previously described [19]. These animals were mated with FVB/N mice for at least 12 generations to obtain Tie2-cre and KLF2^fl/fl^ animals in an FVB/N genetic background. FVB/N Tie2-cre and KLF2^fl/fl^ animals were then mated with each other to obtain Tie2-cre, KLF2^fl/+^ which were consequently mated with KLF2^fl/fl^ animals to obtain Tie2-cre, KLF2^fl/fl^ animals in the FVB/N background. These animals are designated FVB/N Tie2-cre KLF2−/−.

### Light and Electron Microscopic Studies

Embryos were prepared for sectioning by fixing in 2% paraformaldehyde (PFA) and 0.25% glutaraldehyde, and embedded in eponate 12. Serial cross-sections of entire E9.5 and E10.5 embryos of 7 and 5 µm, respectively, were obtained using an LKB 2128 Ultramicrotome. Sections were stained with Toluidine Blue, and images were made using an Olympus BX41 compound microscope and Olympus DP71 digital camera. E9.5 endocardial cell counts were obtained for the central section of the AV canal plus a section 2 sections anterior and a section 2 sections posterior. The ends of the AV canal were designated as 2 endothelial cells beyond the point where the AV canal widens into the ventricle or the atria. E10.5 AV cushion mesenchymal cell counts were obtained for the central section of all sections with endocardial cushion tissue associated with the AV canal. The counts were expressed in cells/mm^2^. Image J 1.46 software was used to trace the irregular outline of the cushion and measure the area. For electron microscopy, embryos were sectioned at 100 nm with an LKB 2128 Ultratome and stained with 5% Uranyl Acetate and Reynold’s Lead Citrate. Images were taken on a JEOL JEM-1230 TEM with a Gatan Ultrascan 4000 digital camera.

### Benzidine Staining

Benzidine staining was performed on cryosections of 4% PFA-fixed FVB/N WT and KLF2−/− embryos. Sections were submerged in 1X PBS for 1 hour prior to benzidine staining as described previously [20]. Sections were then incubated in methanol for 15 seconds, 1% benzidine in methanol for 5 minutes, 2.5% hydrogen peroxide in 70% ethanol for 3 minutes, and washed with DI water for 2.5 minutes.

### Immunohistochemistry

FVB/N KLF2+/− mice were mated to generate E10.5 WT and KLF2−/− embryos. Embryos were fixed in 4% PFA in Millonig’s buffer, washed in 1X Phosphate Buffered Saline Tween-20 (PBST), and incubated in 0.5% hydrogen peroxide, 0.5% normal goat serum (NGS) in PBST. The samples were placed in antibody blocking solution (10% goat serum in PBST) and incubated in primary antibody, PECAM (1∶200) (BD Biosciences). The embryos were incubated with secondary antibody, biotinylated Anti-Rat IgG (1∶500) (Abcam) and BD Pharmingen Streptavidin-Horseradish Peroxidase (Sav-HRP) and BD Pharmingen 3,3′diaminobenzidine (DAB) chromogen in H_2_O_2_ buffer and fixed in 4% PFA. The embryos were frozen and sectioned (10 µm) as described previously [21], [22].

### Alcian Blue Staining

E10.5 WT and KLF2−/− embryos were fixed in a 2% PFA and 0.25% glutaraldehyde solution and cryo-embedded. Cross-sections of 10 µm were cut using a vibratome (Ultrapro 5000). Sections were stained with Alcian Blue (Sigma Aldrich) and Nuclear Fast Red (Sigma Aldrich) and observed using an Olympus BX41 microscope. Images were made using an Olympus DP71 digital camera.

### qRT-PCR Assays

RNA was prepared from E10.5 AV canals, and quantitative RT-PCR (qRT-PCR) was performed as previously described [22]. Some of the primer sequences were as previously described by others: hyaluronan synthase 2 or Has2 [23], Tbx5 [24] Notch1 [25], [26] and Gata4 [24]. The primer sequences for these and the other genes, PECAM1, Ugdh, Sox9 and Tgfβ2, are given in Table S1. Mouse cyclophilin A mRNA was used as an internal standard for normalization. mRNA amounts were quantified using SYBR Green or Taqman reagents (Applied Biosystems). For assays using SYBR Green chemistry, dissociation curves were generated, and it was verified that only one product was amplified. A standard curve from pooled cDNA samples was included in each run to measure the relative amounts of unknown samples. Statistical significance was determined using the Student’s t-test.

### Echocardiography

KLF2+/− adults were mated to obtain E10.5 embryos. Non-invasive *in utero* fetal ultrasound using a VisualSonics Vevo 770 System and 40 MHz mechanical transducer (VisualSonics) was performed on 33 E10.5 embryos. Three pregnancies for the FVB/N and for the mixed genetic backgrounds were examined. The mice were anesthetized using pentobarbital (30 mg/kg; intraperitoneal). The embryos were numbered for genotyping based on their position *in utero*, as described previously [11]. Maternal temperature was maintained using a heat lamp, if required. The 40 MHz VisualSonics RMV transducer gives an axial resolution of 30 µm. For each embryo, blood flow parameters, including heart rate, blood flow velocities and volumes were measured, as previously described [27]. Cardiac output was calculated as stroke volume multiplied by heart rate and expressed as beats per minute. 2D imaging was used to view the 3 or 4 chamber heart and measure left ventricle ejection fraction. To statistically compare the values in WT and KLF2−/−, the Student’s t-test was used.

### AV Explant Collagen Assay

AV regions were dissected from E10.5 FVB/N KLF2−/− and WT hearts and explants were placed on a collagen matrix as previously described [23], [28]–[30]. After a 72 hour incubation, the explants were observed and photographed using an Olympus IX70 inverted microscope with Hoffman Modulation Optics. The cell counts were performed at a single plane of focus, at which the vast majority of the cells could simultaneously be observed.

### Chromatin Immunoprecipitation Assay

ChIP assays were performed essentially as described previously [31], using a KLF2 polyclonal antibody generated using a previously described construct [32], and a negative control preimmune serum. Briefly, for each biological replicate, approximately 3×10^6^ cells from ∼8–10 E10.5 FVB/N WT AV regions were cross-linked with 1% formaldehyde for 10 min at room temperature. Chromatin was sheared to approximately 500 bp using a Bioruptor sonicator (Diagenode, Sparta, NJ). Chromatin was precleared using protein G, precipitated with KLF2 or preimmune antiserum, and cross-links were reversed. DNA was purified and analyzed using quantitative PCR (qPCR) and SYBR Green chemistry. Primer sequences for qPCR are indicated in Table S2. Fold enrichment was calculated as 2^∧^(*Ct*_input_ − *Ct*_test_) and expressed relative to the preimmune serum control.

### Statistical Analysis

The Chi-square test, Student’s t-test or ANOVA were used for statistical analyses, as indicated. Standard deviation was used to measure deviation from the mean, for all experiments. For all of the statistical tests, p values ≤0.05 were considered significant.

## Results

### KLF2−/− Embryos in the FVB/N Genetic Background Die by E11.5

Matings between FVB/N KLF2+/− mice resulted in the expected number of embryos of each genotype at E9.5 and E10.5. Out of 28 embryos from four KLF2+/− matings, no (zero) KLF2−/− E11.5 embryos were obtained (expected frequency = 7). Chi-square analysis was performed to compare the observed and expected frequencies (Table 1), and it was determined that the number of KLF2−/− embryos was significantly less than expected (p = 0.0044). FVB/N KLF2−/− embryos die by E11.5, sooner than KLF2−/−embryos in a mixed genetic background, which die by E14.5 [1], [11], [13]. This suggests that modifier genes in FVB/N affect the KLF2−/− phenotype.

**Table 1:** Number of embryos observed and expected from FVB/N KLF2+/− matings.

Stage	Total viable embryos	WT O	WT E	KLF2+/− O	KLF2+/− E	KLF2−/− O	KLF2−/− E	Chi-square p-value
E9.5	26	8	6.5	12	13	6	6.5	0.7939
E10.5	29	8	7.25	14	14.5	7	7.25	0.9496
E11.5	27	12 (0)	6.75	15 (1)	13.5	0 (2)	6.75	0.0044*

Parentheses indicate number of dead embryos at E11.5. O is observed; E is expected. Asterisk indicates a statistically significant difference between O and E.

### E9.5 FVB/N KLF2−/− Mice have an Accumulation of Cells Lining the AV Canal

**Figure 1:**
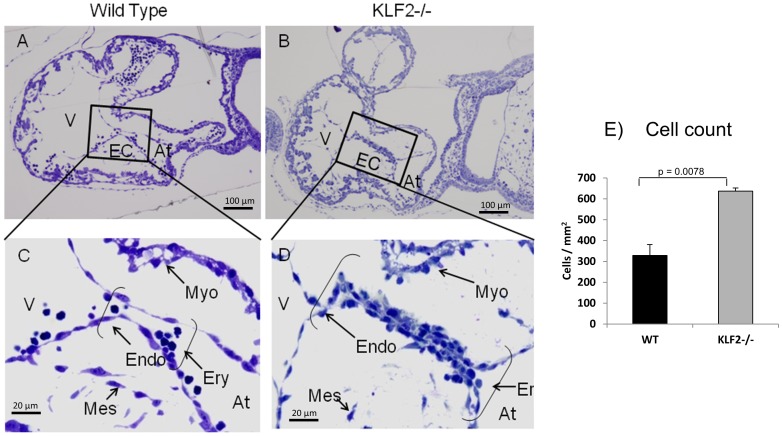
E9.5 FVB/N KLF2−/− atrioventricular cushions have accumulated cells lining the AV canal. (**A**) and (**C**) are WT (n = 4) and (**B**) and (**D**) are KLF2−/− (n = 4) hearts. Micrographs A and B (magnification 50X) show atrial (At) and ventricular (V) chambers. The boxes or brackets enclose the endocardial cushions (EC) shown at higher magnification in C and D (400X). Endo: Endocardial cells; Myo: Myocardium; Ery: erythroid cells; Mes: Mesenchymal cells. (**E**) Bar chart representing the number of cells/mm2 lining the AV canal of E9.5 WT and KLF2−/− embryos. The Student’s t-test indicates that the number of cells is significantly greater in KLF2−/− than in WT (p = 0.0078). n = 4.

In order to investigate the reasons for the earlier embryonic death of KLF2−/− mice in the FVB/N genetic background, serial sections of entire embryos were collected to assess morphological abnormalities. Using light microscopy, anterior to posterior cross-sections of E9.5 FVB/N KLF2−/− embryos were analyzed. Compared to WT littermates, these KLF2−/− embryos appear grossly normal, but at the cellular level there is a dramatic difference in the AV endocardial cushions. In WT embryos, the AV cushions are lined by a single layer of endothelial cells, as expected (Fig. 1A and 1C, n = 3). However in the FVB/N KLF2−/− embryos, there is an increased number of cells lining the AV canal region, and these cells form multiple disorganized layers (Fig. 1B and 1D, n = 3). To determine whether the number of endothelial cells in these E9.5 WT and KLF2−/− atrioventricular cushions is significantly different, cell counts were performed (Fig. 1E). There are 2-fold more cells lining the AV canal in KLF2−/− than in WT. The Student’s t-test indicates that this is a significant difference with a p-value of 0.0078. AV morphological defects were not reported in KLF2−/− hearts from mice in a mixed genetic background [1], [11], [13].

### FVB/N KLF2−/− Embryos have Abnormal Endocardial Cell Morphology

**Figure 2:**
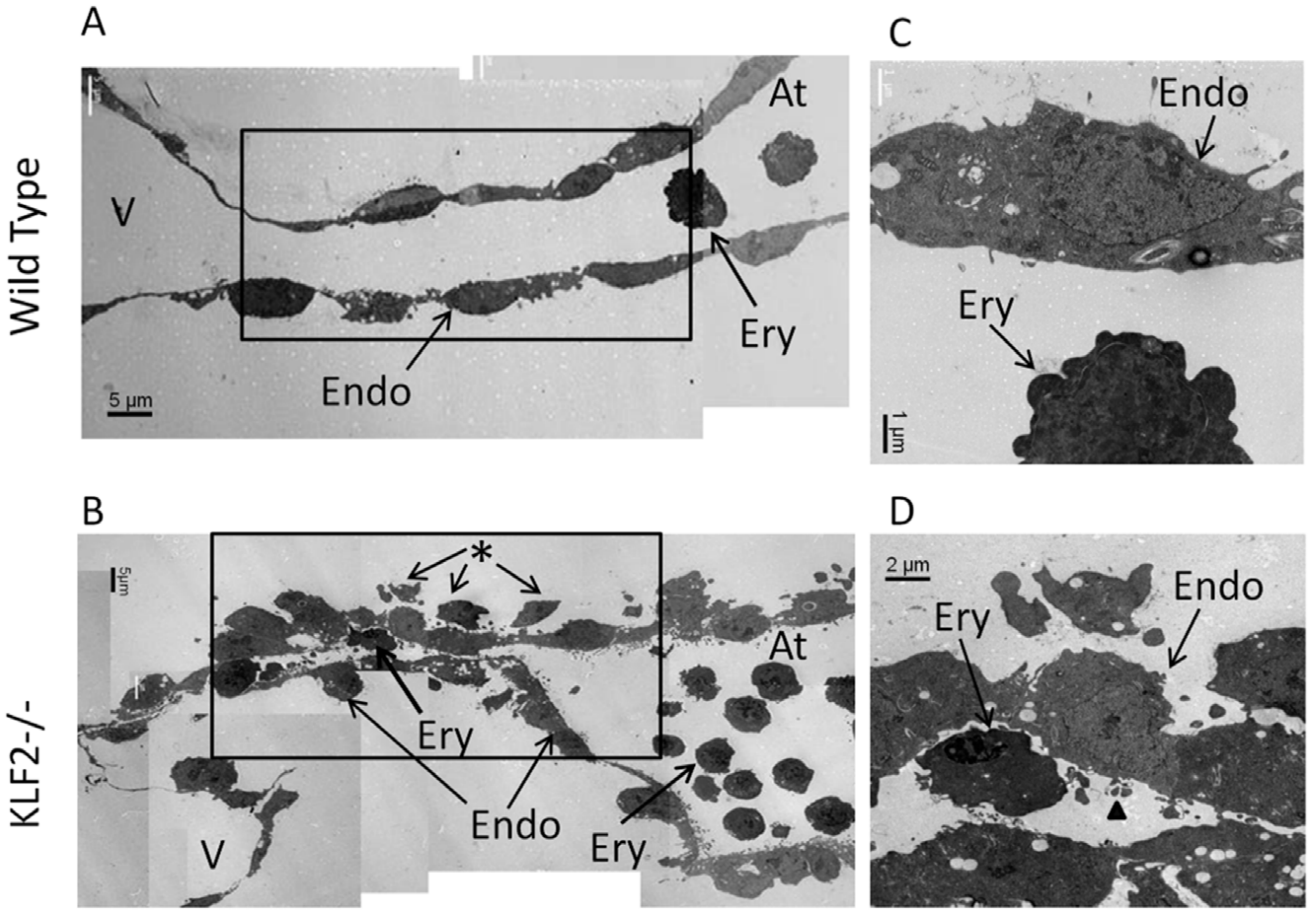
Electron microscopy of E9.5 FVB/N KLF2−/− atrioventricular cushions shows abnormal endocardial cell morphology. In WT (**A**), the endocardium is one cell-layer thick but in KLF2−/− (**B**), there are multiple disorganized cell layers. The boxes enclose the AV canal. At higher magnification, endocardial cells in KLF2 KO (**D**) extend cytoplasmic projections or filopodia-like extensions towards the lumen of the AV canal, which are not observed in WT (**C**). Arrowhead indicates abnormal cytoplasmic projection towards lumen in KLF2−/−. *indicates non-laminar cells accumulated at the lining of the AV canal that are observed in KLF2−/− but not in WT.

The AV cushion region defects in E9.5 FVB/N KLF2−/− mice were studied at the subcellular level using transmission electron microscopy (TEM). TEM reveals that the E9.5 FVB/N WT (Fig. 2A) and the KLF2−/− (Fig. 2B) AV canals are patent and contain erythroid cells. The KLF2−/− AV canal is not lined by typical endocardial cells that are squamous (flat) but instead by cells that are bulbous with numerous cytoplasmic processes extending into the lumen of the AV canal (Fig. 2D). WT cells are squamous and do not have projections or have projections towards the cushions, rather than lumen, as expected (Fig. 2C). Moreover, additional cells lie adjacent to the endocardial layer in KLF2−/− compared to WT, which makes the cushions appear disorganized and non-laminar.

### E10.5 FVB/N KLF2−/− Embryos have Hypoplastic AV Endocardial Cushions and Other Cardiac Abnormalities

**Figure 3:**
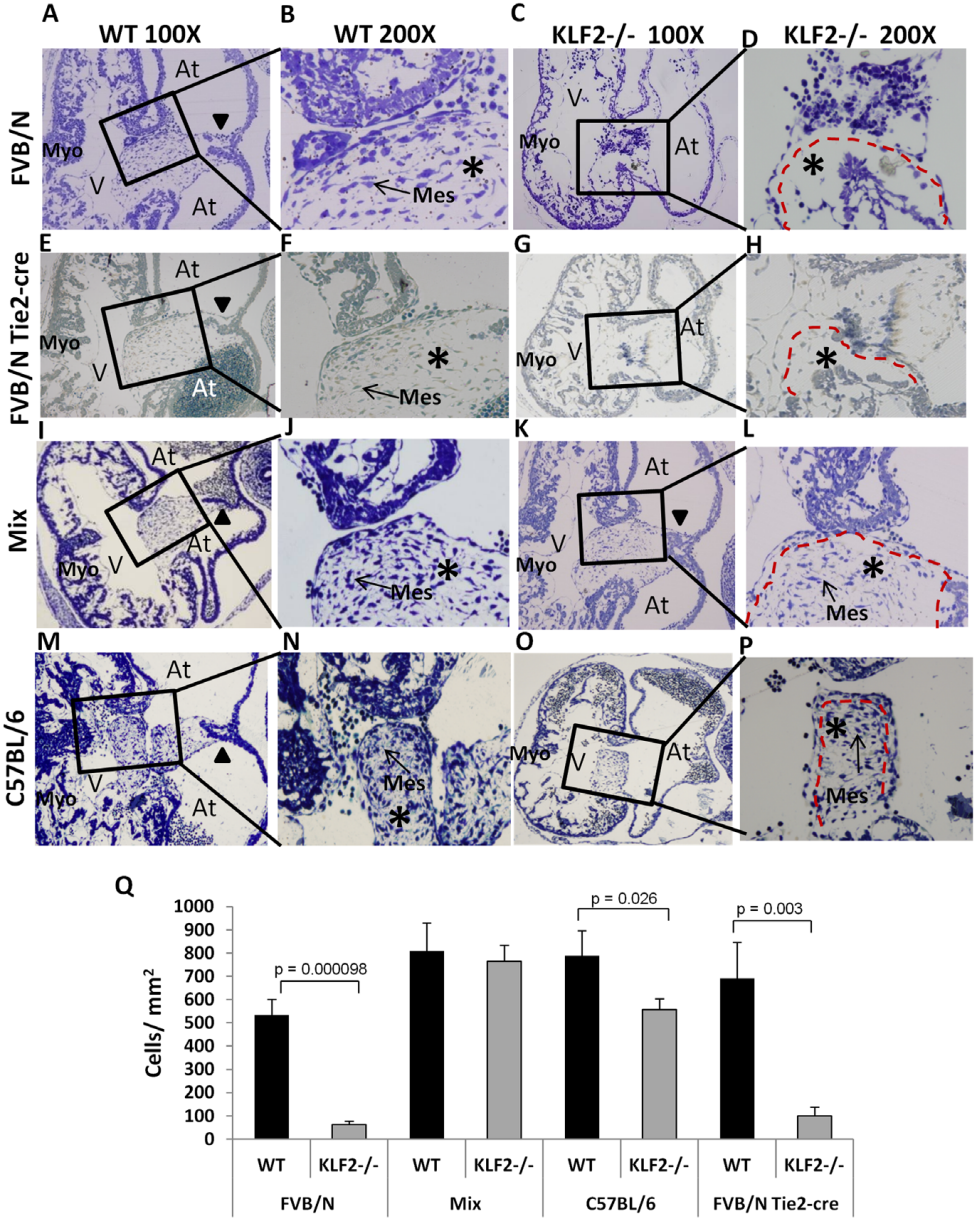
E10.5 FVB/N but not mix and C57BL/6 KLF2−/− atrioventricular endocardial cushions are hypoplastic and disorganized. (**A**) and (**B**) are micrographs of FVB/N WT heart; (**C**) and (**D**) are micrographs of FVB/N KLF2−/− heart. The light micrographs, A and C (magnification 100X), show the structure of the E10.5 heart including the atrial (At) and the ventricular chambers (V) and B and D show the endocardial cushion regions that are in the boxes in A and C respectively, magnified at 200X. The red dashed lines indicate the positions of the hypoplastic FVB/N KLF2−/− AV cushions in D, compared to normal AV cushions in H and L. (**G**) and (**H**) are micrographs of Tie2-cre KLF2−/−, and (**E**) and (**F**) are WT littermate controls without Tie2-cre; all are in the FVB/N background. (**I**) and (**J**) are micrographs of mix WT; (**K**) and (**L**) are micrographs of mix KLF2−/− hearts. (**M**) and (**N**) are micrographs of C57BL/6 WT; (**O**) and (**P**) are micrographs of C57BL/6 KLF2−/− hearts. (**Q**) Bar chart representing the number of mesenchymal cells/mm^2^ in the endocardial cushion tissue associated with the AV canal. Counts were performed of all cells in a single central section from E10.5 WT or KLF2−/− hearts in all three genetic backgrounds and for Tie2-cre KLF2−/−. Student’s t-test indicates that the number of mesenchymal cells is decreased in FVB/N KLF2−/−, Tie2-cre KLF2−/− and C57BL/6 KLF2−/−, compared to WT. Mes: Mesenchymal cells; Endo: Endothelial cells; Ery: Erythroid cells; Myo: Myocardium. n = 3–5 hearts for histological staining, and n = 3 hearts for mesenchymal cell counts. WT and KO embryos for each background are somite matched. Asterisks* indicate AV endocardial cushion region. Arrowheads show atrial septum that is forming in A, E, I, K and M; the atrial septum is absent in C, G and O.

To examine the KLF2−/− AV endocardial phenotype at a later stage of development, light microscopy was performed at E10.5 (n = 3). The cardiac abnormalities in FVB/N KLF2−/− are more severe at E10.5 than at E9.5. The cells in the AV canal region and endocardial cushions in FVB/N KLF2−/− (Fig. 3C and 3D) are disorganized compared to somite matched FVB/N WT controls (Fig. 3A and 3B). In E10.5 FVB/N KLF2−/− embryos, the AV cushions are hypocellular (Fig. 3D at *) compared to FVB/N WT which are highly populated with mesenchymal cells (Fig. 3B at *). In FVB/N KLF2−/−, the endocardial cells are evidently unable to transform into mesenchymal cells and migrate into the cushions, and therefore endothelial-like squamous cells accumulate lining the AV canal. Moreover, the E10.5 FVB/N KLF2−/− heart has only one atrium (Fig. 3C), whereas somite-matched FVB/N WT hearts have a left and right atrium at this time point (Fig. 3A), indicating that there is also an atrial septal abnormality in the septum primum in the mutants. Additionally, in the E10.5 FVB/N KLF2−/− heart (Fig. 3C), the myocardium is thinner than in the FVB/N WT heart (Fig. 3A), as previously reported [11]. KLF2 is not expressed in the myocardium, thus its effect on myocardial development must be indirect.

E10.5 endothelial-specific Tie2-cre KLF2−/− (Tie2-cre KLF2−/−) hearts show hypocellular endocardial cushions, disorganized AV canal regions, and delayed atrial septal formation (Fig. 3G and 3H), similar to the traditional KLF2 KO. Using this model, the deletion of the KLF2 gene in the heart is quite complete at approximately 84% (D. Vinjamur, unpublished data). Negative control littermates having the floxed KLF2 gene without Tie2-cre are unaffected, as expected and shown in Fig. 3E and 3F. This suggests that KLF2 has an endothelial cell-autonomous role in the AV cushion region.

The E10.5 FVB/N KLF2−/− heart morphological phenotype is more severe than that previously reported by Lee et**al. for mice in a mixed genetic background, which had only myocardial thinning [11]. To confirm that the KLF2−/− phenotype varies in different genetic backgrounds, matings were carried out to obtain KLF2−/− embryos in a controlled genetic background that is 50% FVB/N and 50% C57BL/6 (mix KLF2−/−). Light microscopy studies on E10.5 mix KLF2−/− indicate that the AV canal and cushion morphology in these embryos (Fig. 3K and 3L) is comparable to somite- and genetic background-matched mix WT embryos (Fig. 3I and 3J). The endocardial cushions in mix KLF2−/− embryos do not have a drastic reduction in mesenchymal cells (Fig. 3L). However, mix KLF2−/− embryos (Fig. 3K) have thinner myocardium than mix WT (Fig. 3I), as previously reported [11]. C57BL/6 KLF2−/− hearts (Fig. 3O and 3P) have apparently normal AV cushions like mix KLF2−/− and C57BL/6 WT (Fig. 3M and 3N), and thinner myocardium like mix KLF2−/− and FVB/N KLF2−/−. C57BL/6 KLF2−/− hearts (Fig. 3O) have atrial septal abnormality similar to that observed in FVB/N KLF2−/− (Fig. 3C), but not found in mix KLF2−/− (Fig. 3K). To quantify the mesenchymal cell hypocellularity of the AV endocardial cushions, cell counts were performed for E10.5 WT and KLF2−/− embryos in the FVB/N, C57BL/6 and mixed genetic backgrounds, and for the FVB/N Tie2-cre KLF2−/− (Fig. 3Q). As expected, FVB/N KLF2−/− and Tie2-cre KLF2−/− showed highly significant (p<0.001) decreases in the number of mesenchymal cells in the endocardial cushion tissue associated with the AV canal, compared to controls. Interestingly, C57BL/6 KLF2−/− embryos also showed a statistically significant (p = 0.03) decrease in the mesenchymal cell count, suggesting that there is some role for KLF2 in EMT in this strain. There may be unique recessive modifier genes involved in the decreased AV mesenchymal cell numbers in FVB/N and in C57BL/6 KLF2−/− hearts, because the number is normal in mix KLF2−/− hearts.

**Figure 4:**
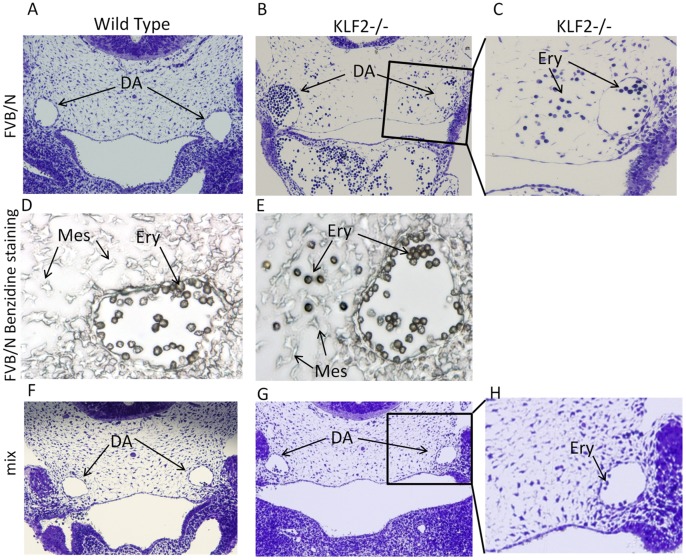
E10.5 FVB/N KLF2−/− but not mix KLF2−/− embryos have erythroid cells outside of the dorsal aortas. (**A**) is a light micrograph (100X) of the FVB/N WT E10.5 dorsal aortas (DA). Erythroid cells (Ery) are found only within the aortas. (**B**) FVB/N KLF2−/− have a number of erythroid cells in tissue surrounding the DA. The box indicates the region that is magnified in C (200X). (**C**) Erythroid cells within and outside the vessel in FVB/N KLF2−/−. (**D**) and (**E**) are light micrographs of benzidine-stained FVB/N WT and KLF2−/− sections, respectively (400X). Ery indicates benzidine-positive, brown-colored erythroid cells. Mes indicates mesenchymal cells. (**F**), (**G**) and (**H**) are mix WT (100X), mix KLF2−/− (100X) and mix KLF2−/− (200X), respectively, showing normal aortas with erythroid cells within the vasculature only. (n = 3 each).

Unlike WT (Fig. 4A), in FVB/N KLF2−/− embryos, erythroid cells are observed outside of the dorsal aortas, suggesting possible hemorrhaging (Fig. 4B and 4C). Alternatively, the presence of erythroid cells outside of the FVB/N KLF2−/− dorsal aortas may be due to an inability of hematopoietic progenitors, originating in the para-aortic mesenchyme, to reach the aortic endothelium and enter circulation [33]. To confirm that these cells are erythroid, benzidine staining was performed. In FVB/N KLF2−/− embryos, there are benzidine-positive cells in the tissue surrounding the dorsal aorta (Fig. 4E), but they are found only within the dorsal aorta in FVB/N WT (Fig.4D). These results were replicated in E10.5 FVB/N WT and KLF2−/− hearts at the 34 and 36 somite stages. There are no blood cells in the tissues surrounding the dorsal aortas in mix KLF2−/− embryos (Fig. 4G and 4H), which look comparable to mix WT (Fig. 4F) and FVB/N WT (Fig. 4A).

The outflow tract endocardial cushions in two out of three E10.5 FVB/N KLF2−/− embryos examined had an abnormal, non-laminar accumulation of endocardial cells lining the lumen. These same embryos also had a smaller number of mesenchymal cells in the outflow tract cushions than WT (Fig. S1). Therefore, although the outflow tract phenotype is not completely penetrant, there are similarities between the AV and outflow tract phenotypes in FVB/N KLF2−/− mice.

### Cells Accumulated in the FVB/N KLF2−/− AV Canal have Endothelial Character

**Figure 5:**
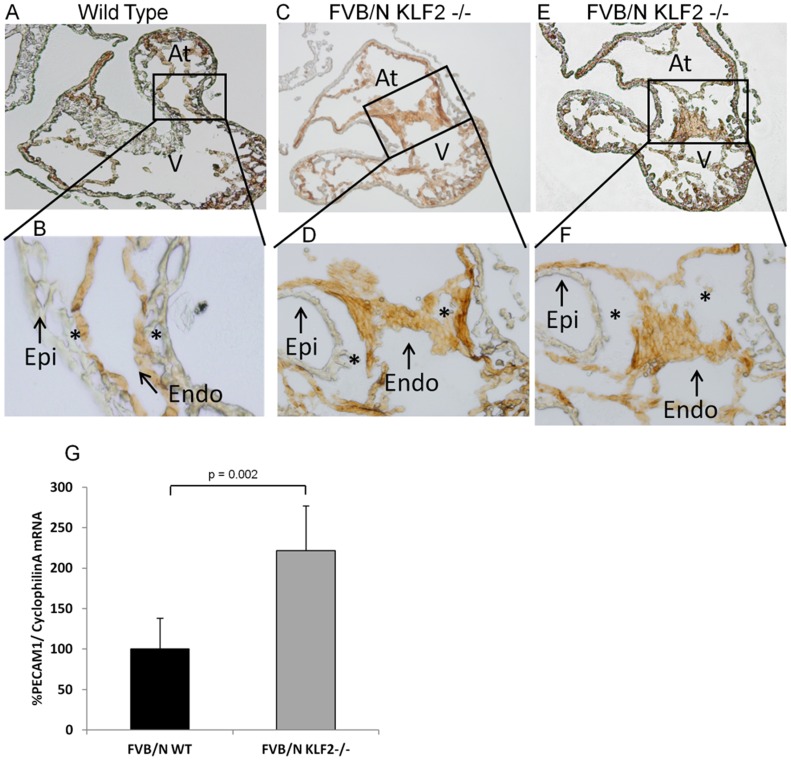
E10.5 FVB/N KLF2−/− hearts have accumulated endothelial cells lining the AV canal. Immunohistochemical staining (n = 2) was performed using an endothelial cell specific mouse PECAM (CD31) antibody. A goat anti-mouse secondary antibody conjugated with HRP was used, and reacted with diaminobenzidine (DAB) for detection. Brown coloration of cells indicates a CD31 positive cell type. (**A**) The cells lining the AV canal in FVB/N WT are CD31 positive. (**B**) Higher magnification of WT AV region. (**C**) and (**E**) Cells accumulated in the AV canal in two different FVB/N KLF2−/− hearts are CD31 positive, indicating that they are endothelial cells. (**D**) and (**F**) Higher magnifications of KLF2−/− AV regions show accumulation of stained cells. Asterisks* indicate AV endothelial cushion region. A, C and E are 100X magnification; B, D and F are 200X. (**G**) qRT-PCR shows a 2-fold increase in expression of PECAM mRNA in FVB/N KLF2−/− AV canals compared to FVB/N WT (p = 0.002, n = 7). The amount of PECAM mRNA in WT was designated 100%.

To further define the role of KLF2 in EMT, the cells accumulating in the FVB/N KLF2−/− AV canal were studied. Immunohistochemical staining was performed using the endothelial specific PECAM (CD31) antibody. The cells accumulated in the FVB/N KLF2−/− AV canal are CD31 positive, indicating that they have endothelial characteristics (results from two embryos shown in Fig. 5C–5F). The FVB/N WT positive control has an organized layer of CD31 positive endothelial cells lining the AV canal (Fig. 5A and 5B). A negative control, reacted only with secondary antibody, had no staining. Expression of PECAM1 mRNA was significantly higher in FVB/N KLF2−/− than in FVB/N WT AV canals as shown by quantitative reverse transcriptase-PCR (qRT-PCR, Fig. 5G). This suggests that there is an increased number of cells expressing PECAM1mRNA, and/or PECAM1 expression per cell is increased in KLF2−/−. However, because an accumulation of endothelial-like squamous cells lining the AV canal is observed in KLF2−/−, there is a high probability that there are a higher number of cells expressing PECAM1 mRNA. These findings support the premise that there is abnormal EMT in the AV cushions of FVB/N KLF2−/− embryos and that the cells abnormally accumulating at the AV canal are endothelial cells that are unable to transform.

### E10.5 FVB/N KLF2−/− Hearts have Reduced Glycosaminoglycans in the Extracellular Matrix

**Figure 6:**
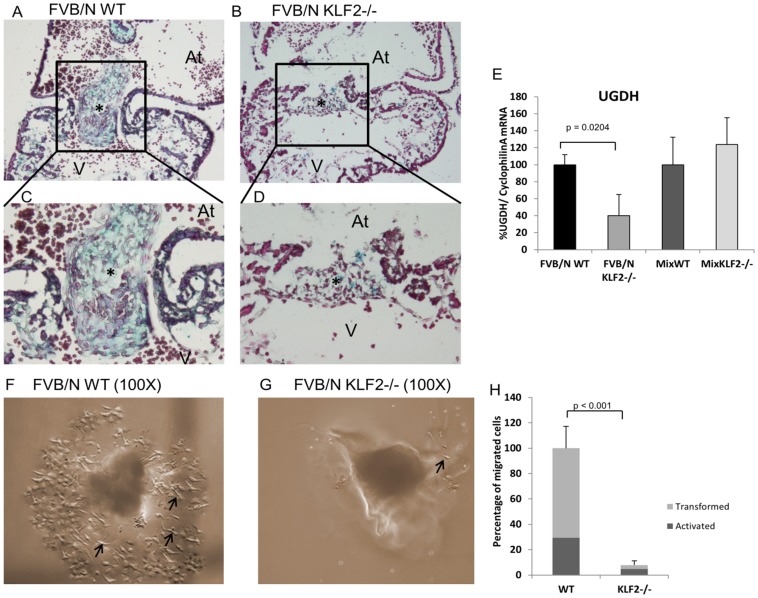
E10.5 FVB/N KLF2−/− hearts have abnormal cardiac jelly, and a reduction in transformed mesenchymal cells. Alcian blue staining for extracellular matrix and counterstain with nuclear fast red was performed on cross-sections of E10.5 FVB/N WT and KLF2−/− hearts (n = 3). (**A**) and (**C**) are WT AV cushions; the nuclei are stained red and the extracellular matrix is stained blue (100X and 200X magnifications, respectively). (**B**) and (**D**) are FVB/N KLF2−/− AV cushions with decreased blue staining, indicating reduced glycosaminoglycans (100X and 200X magnifications, respectively). At: Atrium; V: Ventricle. Boxes indicate the AV endocardial cushion region. (**E**) qRT-PCR indicates a 2-fold decrease in expression of Ugdh mRNA (p = 0.0204) in FVB/N KLF2−/− but not mix KLF2−/− compared to WT AV region (n = 7). (**F**) and (**G**) AV canal explants were incubated *in vitro* on a collagen matrix for 72 hours, and the cells migrating into the matrix were observed (100X magnification). (**F**) FVB/N WT explants show mesenchymal cells migrating into the collagen matrix (n = 5). Arrows indicate mesenchymal cells. Round cells are activated but not transformed. Stellate cells are activated and transformed into mesenchymal cells. (**G**) FVB/N KLF2−/− explants have less mesenchymal cells migrating into the collagen matrix than WT (n = 5), indicating an EMT defect in the FVB/N KLF2−/− endocardial cells. (**H**) The bar chart indicates percentage of transformed cells in FVB/N WT and FVB/N KLF2−/− (n = 3). The number of cells in WT is designated as 100%. KLF2−/− explants have a greater than 10-fold decrease in mesenchymal cells in the collagen matrix compared to WT (p<0.001).

The accumulation of endothelial-like cells in the AV canal and the absence of mesenchymal cells in endocardial cushions in FVB/N KLF2−/− mice suggests that KLF2 regulates EMT during AV cushion formation. Cardiac jelly is a prerequisite for endothelial cell transformation and migration. Glycosaminoglycans form the major component of the cardiac jelly and are essential for endocardial cushion EMT. Therefore, alcian blue was used to stain glycosaminoglycans in WT and KLF2−/− embryos in the FVB/N and mixed genetic backgrounds. At E10.5, FVB/N KLF2−/− hearts have a vast reduction of glycosaminoglycans in the cardiac jelly (Fig. 6B and 6D) compared to WT (Fig. 6A and 6C). This complements the data indicating that KLF2 is required for EMT in the endocardial cushions. Alcian blue staining appears normal in mix KLF2−/− hearts (Fig. S2), indicating that the reduction of GAGs is specific to FVB/N KLF2−/−. To examine whether KLF2 ablation results in reduced expression of genes encoding enzymes involved in the synthesis of glycosaminoglycans, quantitative RT-PCR was performed using RNA from WT and KLF2−/− AV canals. As shown in Fig. 6E, the expression of UDP-glucose dehydrogenase (Ugdh) mRNA was significantly reduced in FVB/N KLF2−/− compared to FVB/N WT, but similar amounts were expressed in mix WT and mix KLF2−/−. This correlates with the reduced alcian blue staining in FVB/N KLF2−/−, but not in mix KLF2−/−, compared to WT. UGDH converts UDP-glucose to UDP-glucuronic acid, which is required for the biosynthesis of GAG components like hyaluronan, heparin sulfate and chondroitin [34]. Expression of the hyaluronan synthase 2 (Has2) gene was not reduced in FVB/N KLF2−/− compared to WT AV canals (data not shown). Therefore a reduction in Has2 is not likely to be the cause of the reduced GAGs observed in FVB/N KLF2−/− hearts.

### A Reduced Number of Mesenchymal Cells Invade the Collagen Gel in FVB/N KLF2−/− AV Explant Assays

To determine whether the EMT defect is caused solely by a defect in the cardiac jelly, or also by an abnormality in the endocardial cells in FVB/N KLF2−/− hearts, E10.5 AV explant assays were performed using a collagen gel. FVB/N WT explants underwent EMT and the mesenchymal cells migrated into the collagen matrix during the 72 hour incubation (Fig. 6F). Compared to WT explants, KLF2−/− explants showed a significant reduction in the number of mesenchymal cells that migrated into the collagen matrix (Fig. 6G and 6H). The KLF2−/− explants have at least a 10-fold reduction in the number of transformed cells compared to WT (Fig. 6H). In addition, the fraction of migrated cells that are transformed is greater than activated cells in WT explants, but the reverse is true in KLF2−/−. This suggests that a defect in the endocardial cells, as well as in the cardiac jelly composition, is responsible for abnormal EMT in the FVB/N KLF2−/− AV cushions.

### FVB/N KLF2−/− Hearts Exhibit Abnormal Cardiac Function

Echocardiography was performed on E10.5 FVB/N WT, FVB/N KLF2−/−, mix WT and mix KLF2−/− embryos. The heart rate was not significantly different in embryos of the 4 genotypes (Fig. S3A), but cardiac output and ejection fraction are significantly higher in FVB/N KLF2−/− than in WT embryos (Fig. S3B and S3C left side, respectively). The descending aorta velocity in FVB/N KLF2−/− hearts is significantly lower than WT (Fig. S3D left side). Higher than normal cardiac output and ejection fraction were observed in E11.5 KLF2−/− embryos in a mixed genetic background [11], but these features were not evident in E10.5 mix KLF2−/− (Figs. S3B, S3C and S3D; right side). It is difficult to reconcile the specific abnormalities in the heart parameters in FVB/N KLF2−/− with the observed morphological defects.

### Cardiovascular Genes Important for AV Cushion Development and Septation are Downregulated in FVB/N KLF2−/− Compared to WT AV Regions

KLF2 is a transcription factor expressed in endothelial cells. To begin to elucidate the molecular mechanism by which KLF2 ablation results in heart development and septation abnormalities, the amounts of expression of candidate genes important in AV cushion development (Tbx5, Sox9, Tgfβ2, Notch1, Gata4) and atrial septation (Tbx5, Gata4) (Reviewed in [7]) were quantified in AV regions dissected from FVB/N WT and KLF2−/− hearts. Each of these genes has at least one consensus KLF2 binding site in its promoter (CCACCC and CCGCCC) [10], [32], within 500 bp upstream of the transcription start site (Table S3).

**Figure 7:**
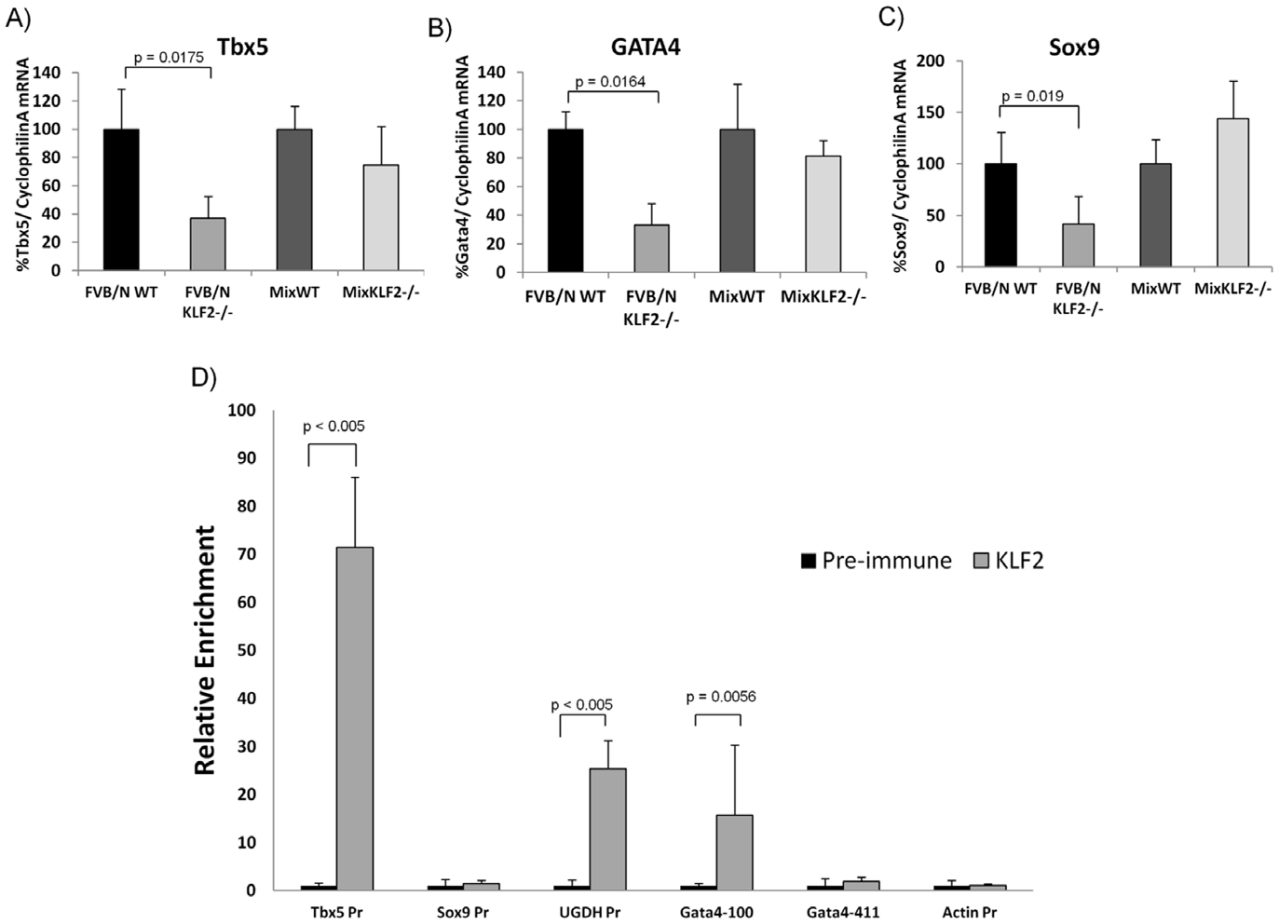
Cardiovascular genes are dysregulated in FVB/N KLF2−/− AV region. Quantitative RT-PCR (qRT-PCR) of AV region RNA was used to test the amount of expression of genes important for AV cushion development (Tbx5 and Sox9) and atrial septation (Tbx5 and Gata4). Cyclophilin A mRNA was used as a normalization control. FVB/N WT and mix WT were designated as100%, and KLF2−/− were compared to WT. (**A–C**) In E10.5 FVB/N KLF2−/− AV region there is significantly decreased expression of (**A**) Tbx5 (p = 0.0175), (**B**) Gata4 (p = 0.0164) and (**C**) Sox9 (p = 0.019) mRNA compared to FVB/N WT, but Mix WT and Mix KLF2−/− hearts have no differences in expression of these genes. Error bars indicate standard deviation. n = 5. **D**) ChIP assays were performed on cells obtained from E10.5 WT AV regions. Approximately 8 to 10 WT AV regions were pooled to obtain cells for one replicate. Polyclonal anti-KLF2 and non-specific control pre-immune serum was used. The y-axis represents the relative fold-enrichment. The mean pre-immune enrichment was designated as ‘1.0’ and the enrichment with KLF2 antisera was scaled appropriately. The x-axis indicates the location of the primers used for qPCR; all were in gene promoters and are described in Table S2. Pr: Promoter. Primers specific for the β-actin gene were used as a negative control. n = 7 biological replicates.

The mRNAs for three cardiovascular transcription factors, Tbx5, Gata4 and Sox 9, showed significantly reduced expression in FVB/N KLF2−/− compared to WT AV regions (Figs. 7A–7C), indicating that these genes are, directly or indirectly, positively regulated by KLF2. FVB/N Tie2-cre KLF2−/− AV canals show a similar and significant reduction in the expression of the Tbx5, Gata4 and Sox9 genes, compared to controls without Tie2-cre (Fig. S4). Interestingly, expression of these genes is not reduced in mix KLF2−/− compared to mix WT, consistent with the absence of the AV cushion and atrial septal phenotypes in mix KLF2−/−. Apparently, strain-specific modifier genes differentially affect the expression of Ugdh, Sox9, Tbx5 and Gata4 mRNA in response to KLF2 ablation. Other roles of Gata4 and Tbx5 are discussed in detail later, but Gata4 and Tbx5 double heterozygous knockout mice show myocardial thinning [24], [35], [36], like KLF2−/−. Mix KLF2−/− has myocardial thinning but no reduction of Gata4 and Tbx5 mRNA, indicating that an additional unknown gene(s) is related to this phenotype. Sox9 plays an important role during endocardial cushion EMT as well as valve remodeling [37]. The Notch1 and Tgfβ2 genes are important for atrioventricular development, but these mRNAs are not expressed significantly differently in KLF2−/− and WT hearts, whether the animals are in an FVB/N or a mixed genetic background (data not shown). The data suggest that Tbx5, Gata4, Sox9 and Ugdh are downstream of KLF2 and have a genetic background specific role in AV endocardial cushion EMT, atrial septation and cardiac jelly synthesis. More than one cardiovascular gene shows reduced expression by 2–3 fold in the absence of KLF2, suggesting that the KO phenotype is an outcome of dysregulation of multiple downstream targets of KLF2.

### KLF2 Binds to the Mouse Gata4, Tbx5 and Ugdh Promoters

KLF2 positively regulates mRNA expression of Gata4, Tbx5, Sox9 and Ugdh. To better understand the mechanism of KLF2 regulation, chromatin immunoprecipitation (ChIP) assays using a KLF2 polyclonal antibody [31] were performed using cells from E10.5 FVB/N WT mouse AV regions. Due to the limited availability of tissue per sample, between eight and ten E10.5 AV regions were pooled for each ChIP assay. The Gata4, Tbx5 and Sox9 promoters have multiple potential KLF2 binding sites, and the Ugdh promoter has a single site (Table S3). Due to assay limitations, the two Tbx5 and two Sox9 binding sites could not be distinguished from each other, and were tested simultaneously. Two regions of the Gata4 promoter, each containing two putative KLF2 binding sites (designated −100 and −411), were tested for KLF2 enrichment. Quantitative PCR (qPCR) was used to determine the fold-enrichment of KLF2 at each promoter region, by comparing ChIP assays with KLF2-antiserum to pre-immune serum. The data in Figure 7D indicate that KLF2 showed an approximately 70-fold enrichment at the Tbx5 promoter, 25-fold enrichment at the Ugdh promoter, and 15-fold enrichment at the proximal Gata4 promoter site (−100) compared to negative control assays using pre-immune serum. No significant KLF2 enrichment was observed at the Sox9 promoter. As a negative control, KLF2 does not bind to the promoter of the β-actin gene. The ChIP assays thus indicate that KLF2 binds to the Gata4, Tbx5 and Ugdh promoters, and therefore may directly regulate these genes. No evidence was obtained to indicate that KLF2 directly regulates the Sox9 gene.

## Discussion

Cardiovascular development and morphogenesis is a complex process, involving a number of highly conserved transcription factors and signaling pathways [38]. KLF2 plays a multi-faceted role in cardiovascular development. It is expressed in the endocardium of the developing heart. The accumulation of endothelial-like cells lining the AV canal, reduced EMT, delayed atrial septal formation, and the absence of normal cardiac jelly composition are novel phenotypes for FVB/N KLF2−/− mice, and therefore may be related to the earlier embryonic death in the FVB/N genetic background. Our studies also identify putative effectors downstream of KLF2 that may impact each of these processes in the embryonic heart.

KLF2 is important for vascular integrity [17]. Kuo *et al.* [1] and Wani *et al*. [13] reported hemorrhaging in the abdominal and cardiac outflow tract region in KLF2−/− embryos. To the contrary, no hemorrhaging was observed in the KLF2−/− embryos examined by Lee et al. [11]. This discrepancy may be partially explained by the current study. Erythroid cells were observed outside E10.5 KLF2−/− dorsal aortas in FVB/N but not in mixed genetic background embryos, indicating that this phenotype is genetic background-specific. The genetic background of the KLF2−/− mice used in the previous studies was not well defined. It is theoretically possible that the dorsal aorta defect in FVB/N KLF2−/− embryos causes shear stress, resulting in the AV cushion defect. However, this hypothesis is not favored because the AV cushion defect is observed by E9.5, whereas the dorsal aorta defect is first apparent at E10.5.

The importance of genetic background in cardiac development has been demonstrated in a number of studies. Sakata et al. studied Hey2 deficient mice and observed a spectrum of cardiovascular anomalies that varied in the BALB/c and C57BL/6 genetic backgrounds [39]. Astrof et al. studied the role of fibronectin in heart development; a null mutation in the gene results in arrested heart development earlier in 129S4 than in C57BL/6 embryos [40]. The current study shows that the role of KLF2 in the morphology and function of the developing heart is also genetic background specific. In the FVB/N background, loss of KLF2 results in an EMT defect in the AV cushion region, delayed formation of the atrial septum, myocardial thinning and death by E10.5. In the C57BL/6 background, KLF2−/− shows delayed atrial septation and myocardial thinning. In a mixed background the major defect in KLF2−/− hearts is myocardial thinning [11].

In this work, we have demonstrated that KLF2 binds the promoters of, and positively regulates, the Tbx5 and Gata4 genes in the mouse E10.5 AV region. An endocardial specific Gata4 KO has multiple layers of endocardium in the AV canal, and hypocellular AV cushions at E10.5 [41], similar to FVB/N KLF2−/−. Tbx5 KO embryos have hypoplastic endocardial cushions [42], like FVB/N KLF2−/−. These phenotypes correlate with our observation that Gata4 and Tbx5 expression is reduced 3-fold in the absence of KLF2 in FVB/N AV canals. Interestingly, the Tbx5 and Gata4 proteins physically interact during cardiac development. A heterozygous mutation (mG295S) in the Gata4 gene disrupts these protein interactions, resulting in cardiac defects like atrial septal defects (ASD), AV septal defects (AVSD) and myocardial thinning beginning at E11.5 [35], [36]. The AVSD and ASD in these mice are known to result from abnormal EMT and remodeling of endocardial cushions. Gata4 mG295S is a missense mutation resulting in diminished DNA binding affinity and transcriptional activity, making it similar to a null allele. In mice with this Gata4 mutation and a null allele for Tbx5, Gata4+/−Tbx5+/−, there is normal EMT but defective remodeling, resulting in septal defects [36]. Like these double heterozygotes, FVB/N KLF2−/− embryos have about 50% less Gata4 and Tbx5 mRNA than normal, and similarly remodeling is affected. However, EMT is affected, indicating that the KLF2−/− embryos are more severely affected than Gata4+/−Tbx5+/−, likely because KLF2 also controls other cardiac genes.

KLF2 binds to and positively regulates the UDP-glucose dehydrogenase (Ugdh) gene. UGDH is expressed in the endocardium and catalyzes conversion of UDP-Glucose to UDP-Glucuronic acid (UDP-GA) [43]. UDP-GA is further converted to hyaluronic acid and other glycosaminoglycans by hyaluronan synthase 2 (Has2) [44]. There is an approximately 2-fold reduction in Ugdh mRNA in the FVB/N KLF2−/− AV canal. This may result in decreased production of UDP-GA and consequently reduced glycosaminoglycans in the cardiac jelly. Interestingly, two missense mutations in the Ugdh gene were recently identified in 3 patients with congenital valve defects [45]. These mutations result in structural defects in UGDH that significantly compromise enzyme function [46]. These findings support our hypothesis that reduced UGDH contributes to the cushion defects in KLF2−/− mice.

Sox9 is a cardiovascular transcription factor expressed in endothelial and mesenchymal cells in the endocardial cushion region [47]. In our study, the Sox9 gene is positively controlled by KLF2, but the lack of evidence for KLF2 promoter binding suggests that the regulation is indirect, or controlled by a more distant DNA element. Sox9 KO results in hypoplastic endocardial cushions and abnormal valve formation [37]. A study by Lincoln et al. showed that ablation of Sox9 in endocardial cells results in reduced EMT [47]. These phenotypes are similar to FVB/N KLF2−/−. The reduced expression of Sox9 in KLF2−/− hearts could be attributed to a reduced number of mesenchymal cells in the AV cushion. However, this seems unlikely because Has2 mRNA, which is also expressed in endocardial and mesenchymal cells, does not show reduced expression in KLF2−/− AV regions.

The current study indicates that KLF2 plays an important role in the synthesis of cardiac jelly, AV endocardial cushion EMT and atrial septation, by regulating several important cardiac genes. These genes include but are probably not limited to Ugdh (cardiac jelly), Sox9 (EMT), and Tbx5 and Gata4 (EMT, AV cushion development, and septation). KLF2 influences the development of the AV cushions and atrial septum, and is likely required for normal cardiac function. Future studies of KLF2 variants may be relevant to the better understanding of human heart defects.

## Supporting Information

Figure S1 **Light micrographs of E10.5 outflow tracts. A)** FVB/N WT outflow tract (OFT, 100X magnification) shows mesenchymal cells in the outflow tract endocardial cushion region. **B)** FVB/N KLF2−/− OFT (100X magnification) shows that the endocardial cushions are hypocellular with respect to mesenchymal cells. Mes: Mesenchymal cells; EC: endocardial cushion. Myo: Myocardium. Embryos are somite matched (36 somites). Arrowhead indicates non-laminar endothelial layer in FVB/N KLF2−/−.
(TIF)

Figure S2 **E10.5 mix KLF2−/− AV cushions stain with alcian blue.** Alcian blue staining for extracellular matrix and counterstain with nuclear fast red on cross-sections of mix WT and mix KLF2−/− hearts (n = 3, 200X magnification). **A)** mix WT and **B)** mix KLF2−/− show alcian blue positive AV cushions suggesting normal composition of GAGs in extracellular matrix. At: Atrium; V: Ventricle.
(TIF)

Figure S3 **E10.5 FVB/N KLF2−/− embryos have increased cardiac output, ejection fraction, and reduced descending aorta velocity.** (A) The heart rate is comparable in all 4 genotypes. (B) Cardiac output and (C) ejection fraction are higher in FVB/N KLF2−/− than FVB/N WT embryos (p = 0.005 and p = 0.037, respectively), there is no difference between Mix WT and Mix KLF2−/−. (D) Descending aorta velocity (DAvel) in FVB/N KLF2−/− hearts is significantly lower than WT (p = 0.01). Error bars indicate standard deviation. n = 5.
(TIF)

Figure S4 **Tie2-cre KLF2−/− AV regions have decreased expression of cardiovascular genes, similar to the traditional KLF2 KO.** Quantitative RT-PCR (qRT-PCR) of AV region RNA was used to test the amount of expression of genes important for AV cushion development (Tbx5 and Sox9), cardiac jelly synthesis (Ugdh) and atrial septation (Tbx5 and Gata4). Cyclophilin A mRNA was used as a normalization control. WT (KLF2^fl/fl^, without Tie2-cre) were designated as 100% and Tie2-cre KLF2−/− was scaled appropriately. In E10.5 Tie2-cre KLF2−/− AV regions there is decreased expression of Gata4 (p = 0.0072), Tbx5 (p = 0.006), Ugdh (p = 0.0012) and Sox9 (p = 0.0014) mRNA compared to WT. All animals are in an FVB/N genetic background. Error bars indicate standard deviation. Students’ t-test was used to compare WT and KLF2−/− gene expression. The brackets indicate significant differences in mRNA expression between WT and KLF2−/−. n = 6.
(TIF)

Table S1 **qRT-PCR primer sequences.**
(DOC)

Table S2 **Location and sequence of the potential KLF2 binding sites in the Tbx5, Gata4, Sox9 and UGDH promoters.**
(DOC)

Table S3 **ChIP primer sequences.**
(DOC)
