# Supplementary figures and images for: Krüppel-Like Factor 2 Is Required for Normal Mouse Cardiac Development
Source: PLoS One. 2013 Feb 14;8(2):e54891. doi: 10.1371/journal.pone.0054891 (PMC3573061; doi:10.1371/journal.pone.0054891)

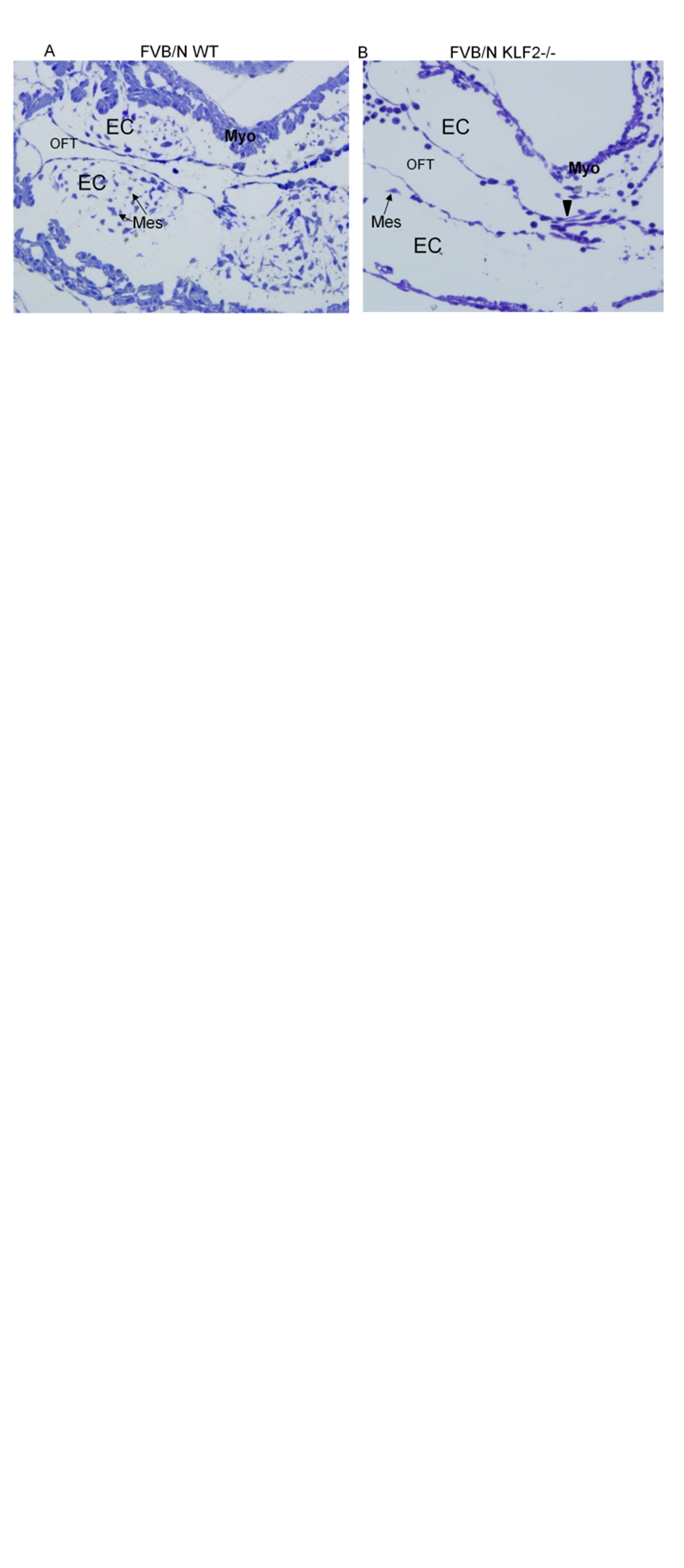

Supplement: Figure S1 — Light micrographs of E10.5 outflow tracts. A) FVB/N WT outflow tract (OFT, 100X magnification) shows mesenchymal cells in the outflow tract endocardial cushion region. B) FVB/N KLF2−/− OFT (100X magnification) shows that the endocardial cushions are hypocellular with respect to mesenchymal cells. Mes: Mesenchymal cells; EC: endocardial cushion. Myo: Myocardium. Embryos are somite matched (36 somites). Arrowhead indicates non-laminar endothelial layer in FVB/N KLF2−/−. (TIF) [file pone.0054891.s001.tif]

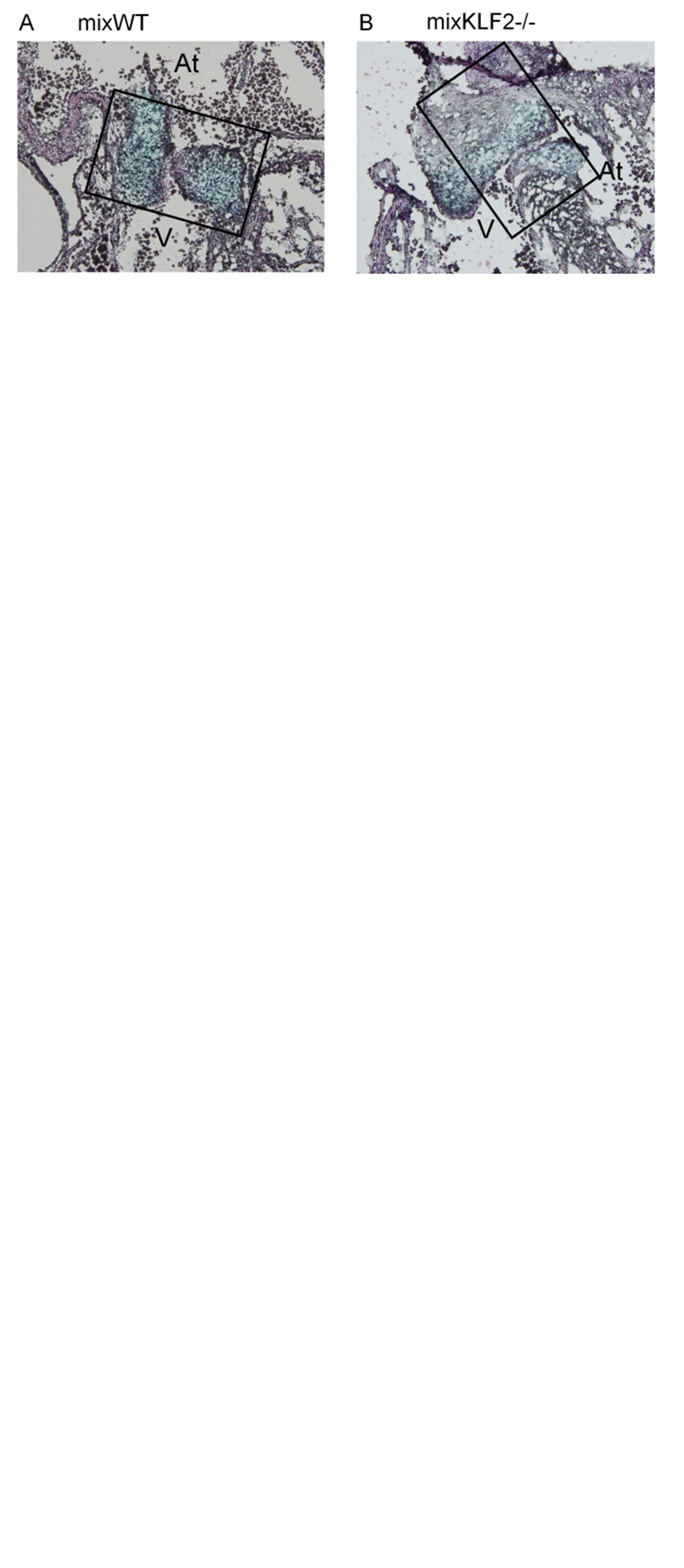

Supplement: Figure S2 — E10.5 mix KLF2−/− AV cushions stain with alcian blue. Alcian blue staining for extracellular matrix and counterstain with nuclear fast red on cross-sections of mix WT and mix KLF2−/− hearts (n = 3, 200X magnification). A) mix WT and B) mix KLF2−/− show alcian blue positive AV cushions suggesting normal composition of GAGs in extracellular matrix. At: Atrium; V: Ventricle. (TIF) [file pone.0054891.s002.tif]

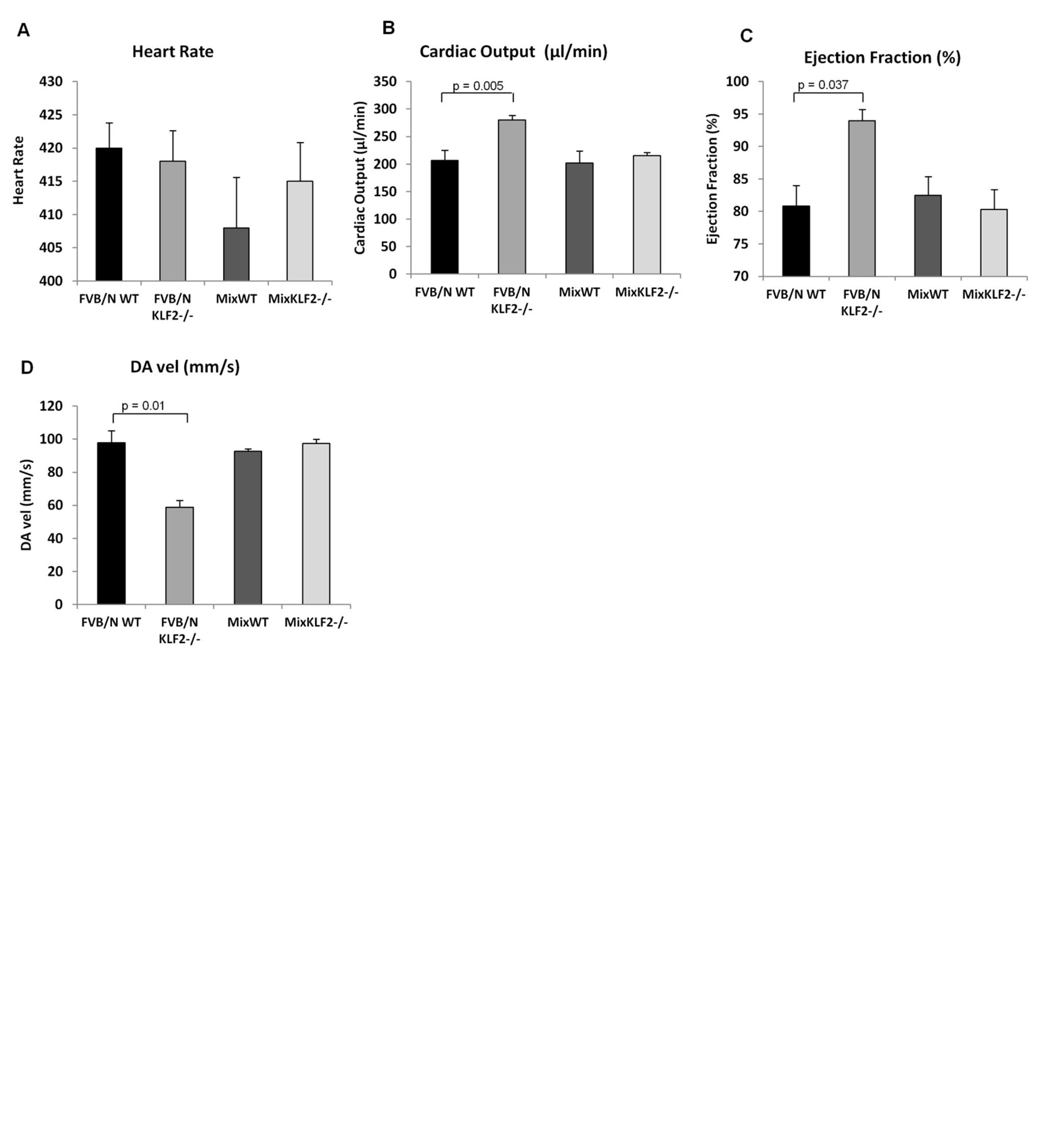

Supplement: Figure S3 — E10.5 FVB/N KLF2−/− embryos have increased cardiac output, ejection fraction, and reduced descending aorta velocity. (A) The heart rate is comparable in all 4 genotypes. (B) Cardiac output and (C) ejection fraction are higher in FVB/N KLF2−/− than FVB/N WT embryos (p = 0.005 and p = 0.037, respectively), there is no difference between Mix WT and Mix KLF2−/−. (D) Descending aorta velocity (DAvel) in FVB/N KLF2−/− hearts is significantly lower than WT (p = 0.01). Error bars indicate standard deviation. n = 5. (TIF) [file pone.0054891.s003.tif]

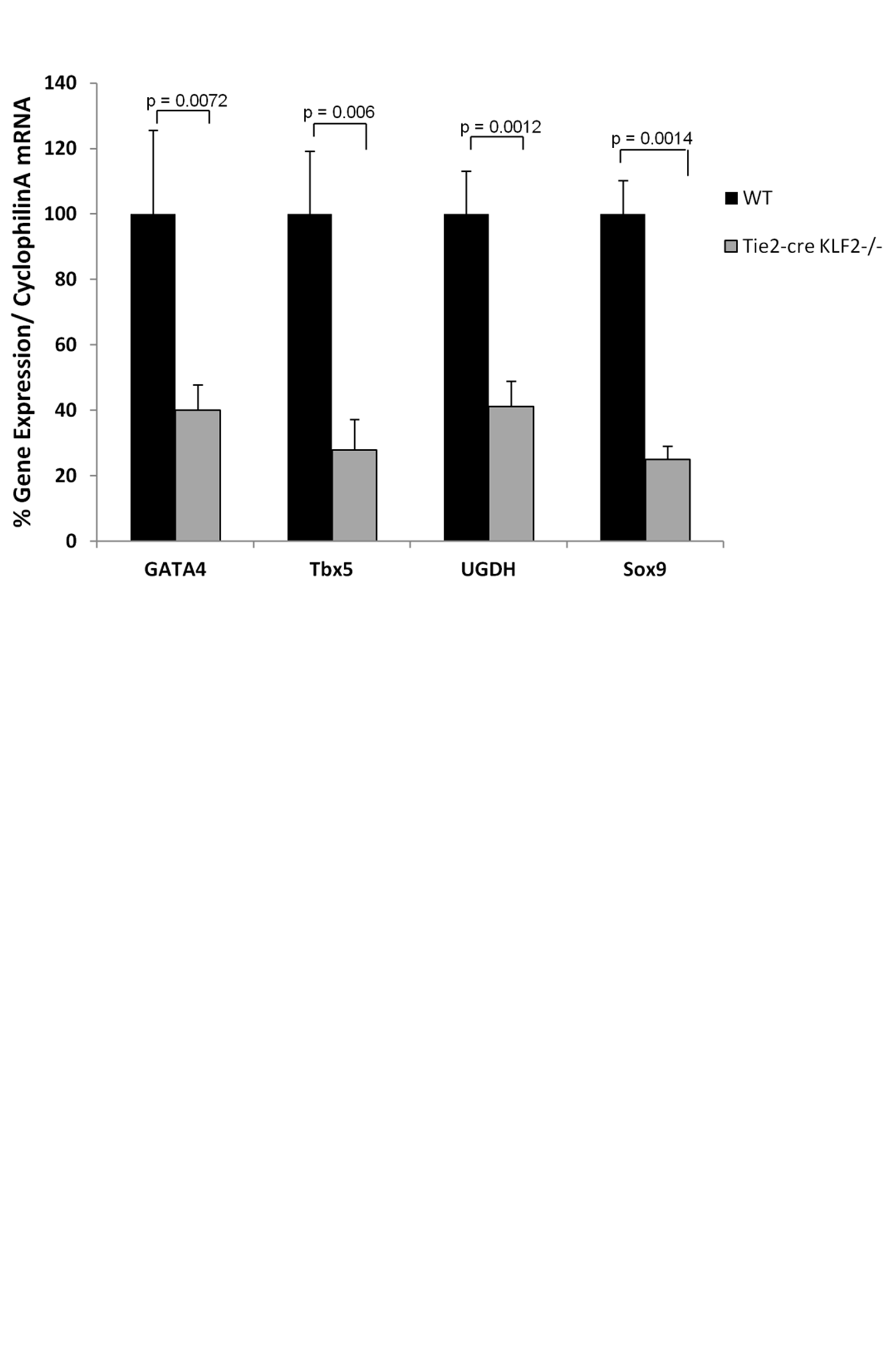

Supplement: Figure S4 — Tie2-cre KLF2−/− AV regions have decreased expression of cardiovascular genes, similar to the traditional KLF2 KO. Quantitative RT-PCR (qRT-PCR) of AV region RNA was used to test the amount of expression of genes important for AV cushion development (Tbx5 and Sox9), cardiac jelly synthesis (Ugdh) and atrial septation (Tbx5 and Gata4). Cyclophilin A mRNA was used as a normalization control. WT (KLF2fl/fl, without Tie2-cre) were designated as 100% and Tie2-cre KLF2−/− was scaled appropriately. In E10.5 Tie2-cre KLF2−/− AV regions there is decreased expression of Gata4 (p = 0.0072), Tbx5 (p = 0.006), Ugdh (p = 0.0012) and Sox9 (p = 0.0014) mRNA compared to WT. All animals are in an FVB/N genetic background. Error bars indicate standard deviation. Students’ t-test was used to compare WT and KLF2−/− gene expression. The brackets indicate significant differences in mRNA expression between WT and KLF2−/−. n = 6. (TIF) [file pone.0054891.s004.tif]
