# Supplementary material for: Krüppel-Like Factor 2 Is Required for Normal Mouse Cardiac Development
Source: PLoS One. 2013 Feb 14;8(2):e54891. doi: 10.1371/journal.pone.0054891 (PMC3573061; doi:10.1371/journal.pone.0054891)
Supplement: Table S1 — qRT-PCR primer sequences. (DOC) [file pone.0054891.s005.doc]

**Table S1: qRT-PCR primer sequences**

| **Gene** | **Forward Primer** | **Reverse Primer** |
| --- | --- | --- |
| Tbx5 | CAAACTCACCAACAACCACC | GCCAGAGACACCATTCTCAC |
| Gata4 | CGAGGGTGAGCCTGTATGTAA | GCTAGTGGCATTGCTGGAGT |
| UGDH | CTGCCCGAAGTAGCTCGTTA | CCTCCTCCTCTGGTAGTCATTC |
| Sox9 | CGGCTCCAGCAAGAACAAG | TGCGCCCACACCATGA |
| Has2 | AGTCATGTACACAGCCTTCAG | CTCCAACACCTCCAACCATAG |
| Notch1 | TGCCACTATGGTTCCTGTAA | GGTTACTGTTGCACTCGTTG |
| TGFβ2 | ATGCCAGTCATCTTGGCC | CGGTGAACTTCCGACTC |
