# Supplementary material for: Krüppel-Like Factor 2 Is Required for Normal Mouse Cardiac Development
Source: PLoS One. 2013 Feb 14;8(2):e54891. doi: 10.1371/journal.pone.0054891 (PMC3573061; doi:10.1371/journal.pone.0054891)
Supplement: Table S2 — Location and sequence of the potential KLF2 binding sites in the Tbx5, Gata4, Sox9 and UGDH promoters. (DOC) [file pone.0054891.s006.doc]

**Table S2: Location and sequence of the potential KLF2 binding sites in the Tbx5, Gata4, Sox9 and UGDH promoters**

| **Gene** | **Location of KLF2 consensus binding site with respect to transcription start** | **KLF2 binding site sequence** |
| --- | --- | --- |
| Tbx5 | -389  -333 | CCGCCC  CCACCC |
| Gata4 | -411  -338, -85, -78 | CCACCC  CCGCCC |
| Sox9 | -172, -250 | CCACCC |
| UGDH | -73 | CCACCC |
