# Supplementary material for: Krüppel-Like Factor 2 Is Required for Normal Mouse Cardiac Development
Source: PLoS One. 2013 Feb 14;8(2):e54891. doi: 10.1371/journal.pone.0054891 (PMC3573061; doi:10.1371/journal.pone.0054891)
Supplement: Table S3 — ChIP primer sequences. (DOC) [file pone.0054891.s007.doc]

**Table S**3: ChIP primer sequences

| **Gene Promoter** | **Forward Primer** | **Reverse Primer** |
| --- | --- | --- |
| Tbx5 | GGAGACAGAAATCGGGTGAG | TTGCAGGGAGGAAAGAAAAA |
| Gata4-100 | AAACACGATCCTTGGCAGAG | GACTGGCCTAAGGGAGTCAC |
| Gata4-411 | CCTTAAGGGCCAGTTCAGGT | CTCTGCCAAGGATCGTGTTT |
| Sox9 | CACACACACACATCGGTTCA | AGACAGGAGGGGAGGAGAAG |
| UGDH | AGCACAGACAAGGATGACCA | GGCAGGCCTCTATTTCCTTC |
